# Validity of an observational assessment tool for multifaceted evaluation of faecal condition

**DOI:** 10.1038/s41598-019-40178-5

**Published:** 2019-03-06

**Authors:** Harumi Ohno, Haruka Murakami, Kumpei Tanisawa, Kana Konishi, Motohiko Miyachi

**Affiliations:** Department of Physical Activity Research, National Institute of Health and Nutrition, NIBIOHN, Tokyo, 162-8636 Japan

## Abstract

Faecal volume, form, colour, and odour are associated with various diseases, dietary habits, and the gut microbiome. Multifaceted assessment of faecal condition will be needed for future research and practice. Faecal observation has advantages, as it is non-invasive, frequent, and easy. We have developed and validated an illustrative card tool for comprehensively faecal assessment. In 38 healthy adults, observations of volume, form, colour, and odour of faeces using the tool were compared to the objective characteristics of the actual faeces determined using a weighing scale, moisture meter, hardness meter, colourimeter, and odour measuring device. A significant positive correlation (ρ = 0.778) was observed between the number of faecal model (2 cm × 10 cm) units and the actual weight. The Bristol Stool Form Scale showed a significant positive correlation with the moisture content (ρ = 0.717) and negative correlations with faecal hardness (ρ = −0.843) and adhesiveness (ρ = −0.761). The L*a*b* colour space values of the stool differed significantly among observational judgments using the colour card tool. No significant correlation was observed between the observation of odour and the measured odour index. In conclusion, the faecal volume, form, and colour can be estimated by observation using the multifaceted assessment card tool.

## Introduction

Human faeces consist of residues and moisture remaining after the absorption of nutrients from food and the bacteria coexisting in the intestine (gut microbiome)^1,2^. The faecal condition is associated with various diseases^3–9^ as well as the state of dietary and nutrient intake^10,11^, and may be reflective of the characteristics of the gut microbiome^12,13^.

A previous study revealed a negative correlation between faecal weight and the risk of developing colon cancer^3^, and this risk was lower if the daily stool volume was above 150 g^14^. Faecal form has also been reported to be associated with disease; patients with irritable bowel syndrome^15^ have been reported to frequently repeat defecation with loose and lumpy faeces^4,16,17^, while patients with urinary tract symptoms or enlarged prostate^18,19^ and gallbladder disease^20^ have faeces with lumpy and loose forms, respectively. Moreover, an association between faecal condition and gut microbiome has also been reported with a higher occurrence of loose and watery stool in individuals whose gut microbiome was classified as the *Prevotella* enterotype^13^. Associations between stool colour and biliary atresia in infants^21^, as well as upper and lower gastrointestinal bleeding^22^, have also been described. The faecal gas in individuals with irritable bowel syndrome, ulcerative colitis, colon cancer, and adenoma may contain specific patterns of volatile compounds^23–26^. Furthermore, a positive correlation between faecal volume and intake of dietary fibre has been reported^27^. Therefore, evaluation of faecal condition based on different parameters may be useful in screening for various diseases and may help to understand the status of an individual’s nutrient intake.

The Bristol Stool Form Scale (BSFS) is a well-known tool for classifying faecal form^28–30^. Stool colour cards have also been used to screen for biliary atresia in infants^21,31–34^ and for gastrointestinal bleeding in adults. However, no tools are available that allow convenient observation and evaluation of faecal volume and odour, and no tools have been reported that allow for comprehensive observation and evaluation of faecal condition. Therefore, this study was performed to develop and evaluate the validity of a multifaceted tool to assess faecal characteristics, such as volume, form, colour, and odour. To our knowledge, this is first validation study of an assessment tool for the observational evaluation of multifaceted faecal condition in healthy adults.

## Methods

### Participants

Overall, 38 adults aged between 22 and 79 years participated in this cross-sectional study (men, *n* = 20; women, *n* = 18). All participants were physically independent and had no history of cancer, cardiovascular, liver, or gastrointestinal disease (colonic disease and gallbladder disease), or diabetes. The participants were also questioned about exposure to antibiotics, laxatives, or anti-flatulence drugs in the previous month. The study protocol was reviewed and approved by the Research Ethical Review Committee of the National Institute of Biomedical Innovation, Health, and Nutrition (approval number: health and nutrition 42-01). Study procedures as well as the risks associated with participation were explained and written informed consent was obtained from all participants. Moreover, all study procedures were performed in accordance with relevant guidelines/regulations.

### Study procedure

This cross-sectional study examined: (1) the validity of observational evaluations by each participant and by one researcher using the faecal assessment tool in comparison to objective and physical evaluations, and (2) the degrees of coincidence of the same faecal evaluation by each participant and the researcher performed using the faecal assessment tool.

The participants arrived at the laboratory according to their daily bowel movement schedule. Following measurements of height, weight, and body fat, the participants rested until they responded to their bowel movement, and defecated the entire faecal amount onto a disposable polypropylene tray (250 × 175 × 31 mm) in the laboratory toilet. After defecation, the faecal weight was immediately measured by the researcher. The participants then used the faecal assessment tool to observe and evaluate the volume, form, colour, and odour of their own faeces. Similarly, the researcher used the same tool to examine these characteristics. After completion of the tool-based evaluation, the whole sample was uniformly mixed. Based on the stool output, 1–5 polystyrene Petri dishes (40 mm in diameter and 10 mm in depth) were each filled with 10 g of the faeces sample to analyse its hardness, colour, and odour. To measure the moisture content, 5–7 g of the sample was placed onto an aluminum plate. All observations and analyses were completed within 1 hour after defecation. All measurements were carried out at a temperature of 21.5 °C ± 1.3 °C and 26.6% ± 10.2% humidity. If participants did not produce a bowel movement within 3 hours after arriving at the laboratory, they were requested to return on another day to repeat the experiment.

### Faecal assessment tool

**Figure 1:**
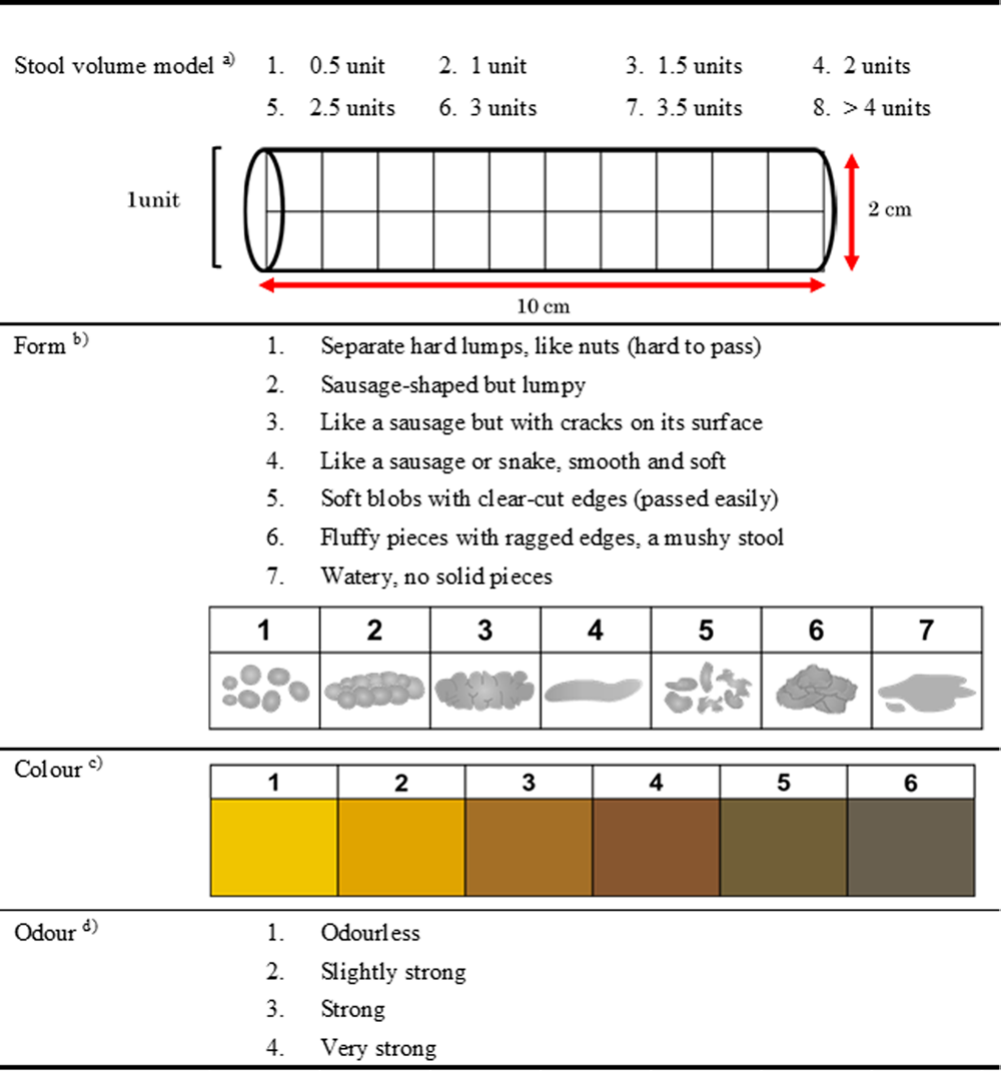
Tool for assessment of faecal volume, form, colour, and odour. (**a**) The faecal amount was estimated by converting the actual volume into a number of faecal model units (i.e., stool model: 2 cm in diameter and 10 cm in length). (**b**) The descriptions are based on the Bristol Stool Form Scale^40^. (**c**) The six colours were based on Munsell’s colour system as described in the JIS colour standard Z 8721: 1, Yellow (5Y 8/8); 2, Light yellowish-brown (2.5Y 7/12); 3, Yellowish-brown (10YR 5/8); 4, Brown (7.5YR4/6); 5, Dark olive brown (5Y 4/4); 6, Dark olive gray (2.5GY 4/3). The colour reproduction may differ from the actual card colours. (**d**) Strength of odour on the day of defecation.

We developed a faecal assessment tool to compare and observe actual faecal volume, form, colour, and odour (Fig. 1). The faecal amount was estimated by the number of stool units (from 1, 0.5 units to 8, >4 units) referring to the stool model (2 cm in diameter, 10 cm in length, cylindrical in shape) illustrated on the handout. A 10-cm scale was also printed on one side of the handout to allow estimation of faeces length and width. The BSFS was designed with written descriptors^28^ but representative pictures of each stool type were subsequently added for clarity. The faeces form was evaluated based on the modified BSFS (Fig. 1): types 1 and 2, hard stool; types 6 and 7, loose/liquid; and types 3, 4, and 5, normal stool^35^. For faecal colour assessment, the closest of the six colours indicated on the faecal assessment tool compared to the actual colour of the faeces was selected. The colour observation was conducted under fixed light conditions (standard fluorescent lamp D65 light source). The six colours on the faecal assessment tool were the colours listed on the colour standard Z8721 (JIS Z 8721-1964): 1) 5Y8/12 (yellow), 2) 2.5Y7/12 (light yellowish-brown), 3) 10YR5/8 (yellowish-brown), 4) 7.5YR7/12 (brown), 5) 5Y4/4 (greenish-dark brown), and 6) 2.5GY4/3 (dark brown). Finally, faecal odour was evaluated on a scale from 1–4 (1, odourless; 2, slight; 3, strong; 4, very strong).

### Objective measurement of faecal characteristics

#### Faecal weight

To evaluate the validity of the faecal model of the faecal assessment tool, the entire stool sample was weighed after defecation using a weighing scale (KD-192; Tanita Co. Ltd., Tokyo, Japan). The weight was then compared to the faecal units on the faecal assessment tool (Fig. 1).

#### Moisture content

Faeces form is associated with its moisture content^36,37^. Therefore, we also examined the relationship between faeces form and moisture content determined using a moisture meter (MB-25; Ohaus Co. Ltd., Parsippany, NJ). Briefly, a 5–7-g faecal sample was uniformly distributed on an aluminum plate and dried at 120 °C. When weight showed a reduction of less than 1 mg during heating for 60 s, the heating process was completed and the dry weight was measured. The moisture content percentage of the faeces was calculated using the following formula: faecal moisture content percentage (%) = (moisture weight − dry weight)/moisture weight × 100%.

#### Hardness and adhesiveness

**Figure 2:**
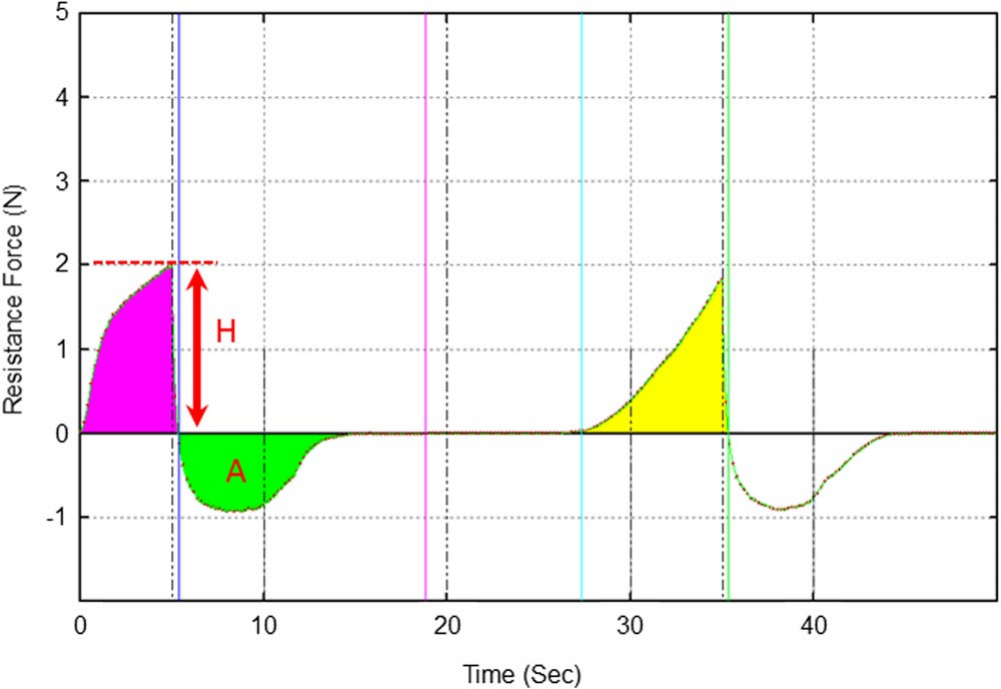
Parameter of faecal hardness and adhesiveness using a Creep Meter. H: Faecal hardness (N); A: Faecal adhesiveness (J/m^3^). The peak weighted value was used for faecal hardness and the area under the curve was used for faecal adhesiveness. The first of the two measurements was used. The yellow area shows the value when pressed the second time, which was not used in data analysis.

To evaluate the potential associations between faecal form, hardness, and adhesiveness, we examined the relationship between the BSFS and hardness and adhesiveness using a Creep Meter viscoelastic measurement device (Rheoner RE2–3305, Yamaden Co. Ltd., Tokyo, Japan). A Petri dish (40 mm in diameter and 10 mm in height) was filled with faecal sample and fixed to the Creep Meter. After adjusting the default position of the cylindrical resin plunger (11.3 mm in diameter and 30 mm in height) (P-16; Yamaden) relative to the surface of the sample, it was compressed to a depth of 5 mm (measurement distortion rate 50%) from the surface of the sample at a compression rate of 1 mm/s. After returning the plunger to 5 mm from the sample surface, this compression was repeated. The texture curve profile parameters were determined as follows (Fig. 2): (1) Hardness (N) was defined as “the maximum force required for compressing the sample” and was calculated as the peak force of the first compression of the sample; (2) Adhesiveness (J/m^3^) was calculated as the integration of the negative force between the first and second compressions. Numerical values were calculated using automatic texture analysis software (TAS-3305, Texture Analyzer for Windows Ver.1.3; Yamaden). A maximum of three faecal samples were measured per participant, and the mean values were used in the analyses.

#### Colour analysis

Faecal colour was measured using a colourimeter (CR-20 Konica Minolta Holdings, Inc., Tokyo, Japan) that can assess the L*a*b* colour space. The L* of the colourimeter represents lightness based on a scale from 0 to 100 with higher values indicating lighter colour (L* = 0 is black and L* = 100 is diffuse white). Red was represented by positive a* values and green was represented by negative a* values, while yellow was represented by positive b* values and blue was represented by negative b* values. The device was calibrated against a standard white plate. The surface of the faeces in the Petri dish was covered tightly with polyethylene cling wrap and the central area of the faecal sample was measured. A maximum of three faecal samples were measured per participant and the mean values were used in the analyses. Each L*, a*, and b* value was compared between the six colours on the faecal assessment tool.

#### Odour

The faecal odour was measured using an odour measuring device (XP-329IIIa; New Cosmos Electric Inc., Osaka, Japan)^38^. This device is highly sensitive but cannot select specific odour components; therefore, the output reading is the result of a composite of various odour components. After the faecal sample in the Petri dish was placed into a sealed glass container (capacity, 800 mL) and odour was allowed to fill the container for 3 minutes, the suction opening of the container lid was connected to the odour measuring device with a Teflon tube. After 1 minute, the sensor reading was recorded. The measurement value was indicated on a scale from 0 to 2000, to represent the intensity of faecal odour. The odour intensity of the air inside an empty container was subtracted from the faecal odour intensity and the result was recorded. A maximum of two faecal samples were measured per participant.

### Statistical analysis

The sample size was calculated using the G*Power statistical power analysis software^39^. Using the correlation coefficient of the faecal form (questionnaire) and bowel transit time (measurement value) of 0.77^40^, the sample size for this study was calculated by setting the verification power to 80% with an effect size of 0.5 (moderate), and a significance level of α = 0.05. Although the smallest calculated sample size was 21 participants, the final number of participants was set at over 30 considering the possibility of insufficient samples or missing data. Continuous variables are expressed as means and standard deviation, while categorical variables are expressed as medians and percentages (%). The relationships between the observational evaluations (volume, form, colour, odour) using the faecal assessment tool and the actual characteristics of the faeces (weight, moisture content, hardness, adhesiveness, colour value, odour intensity) were tested using Spearman’s Rank-Order Correlation. The relationships between the participants’ responses and those of the researcher using the faecal assessment tool were tested using Spearman’s Rank-Order Correlation and weighted κ coefficients. Interpretation of Kappa values given by Landis and Koch (1977). Kappa Agreements were: <0.20, slight; 0.21–0.40, fair; 0.41–0.60, moderate; 0.61–0.80, substantial; and ≥0.81, almost perfect^41^. The comparisons of mean colour values (L*a*b*) were analysed using one-way analysis of variance (ANOVA) and Tukey’s honestly significant difference tests for multiple comparisons. IBM SPSS Statistics for Windows, version 22.0 (IBM, Corp., Armonk, NY) was used for statistical analyses. In all analyses, *P* < 0.05 was taken to indicate statistical significance.

## Results

### Participant characteristics

The mean age of the 38 participants was 43.9 ± 16.4 years, and their physical characteristics were as follows: height 164.3 ± 8.5 cm; weight 61.3 ± 10.4 kg; body mass index, 22.6 ± 2.9 kg/m^2^; and body fat, 23.5 ± 7.4%. None of the participants had taken antibiotics in the month preceding the study, but two participants had taken laxatives and three had taken anti-flatulence drugs 2 weeks before the study.

### Comparison of the observational evaluations performed using the faecal assessment tool and physical and objective evaluations

#### Stool volume

The stool volumes observed by the participants were classified into eight different categories (0.5–4 stool model units) and were uniformly distributed across the entire category range (Table 1). The median stool volume as assessed by the participants was category 3, which was equivalent to 1.5 units in the stool model. Furthermore, 84% of the participants reported stool volumes equivalent to 0.5–2 stool model units.

**Table 1:** Distribution of observed faecal conditions of the participants (*n* = 38).

		*N*	%
Stool volume	1. 0.5 units	9	23.7
	2. 1 unit	7	18.4
	3. 1.5 units	7	18.4
	4. 2 units	9	23.7
	5. 2.5 units	1	2.6
	6. 3 units	3	7.9
	7. 3.5 units	1	2.6
	8. 4 units	1	2.6
Stool form	Type 2	2	5.3
	Type 3	9	23.7
	Type 4	16	42.1
	Type 5	5	13.2
	Type 6	5	13.2
	Type 7	1	2.6
Stool colour	Colour 3	11	28.9
	Colour 4	21	55.3
	Colour 5	6	15.8
Stool odour	1. Odourless	1	2.6
	2. Slightly strong	13	34.2
	3. Strong	20	52.6
	4. Very strong	4	10.5

Values are shown as the number of participants (*n*) or distribution (%).

**Figure 3:**
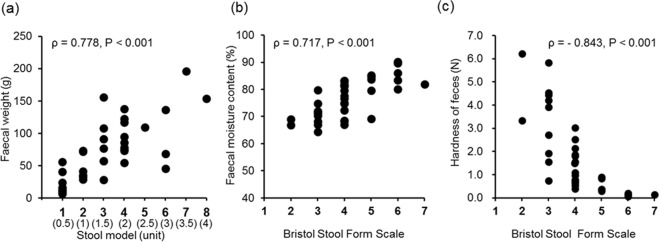
Correlations between observational evaluations (volume, form) using the faecal assessment tool and objective measurement of the actual faeces. (**a**) Correlation between the number of faecal models and the actual weight of the faeces. The number of stool models (cylindrical shape with a diameter of 2 cm and a length of 10 cm) selected by the 38 healthy participants was correlated with the actual weight of the faeces. (**b**) Correlation between the faecal form using the Bristol Stool Form scale and the faecal moisture content. The Bristol Stool Form Scale classified by 38 healthy participants was correlated with the faecal moisture content. (**c**) Correlation between the faecal form using the Bristol Stool Form scale and the hardness of the faeces. The Bristol Stool Form Scale classified by the 38 healthy participants was correlated with the hardness of the faeces.

The measured weight was 69.2 ± 47.0 g (range, 6.4–196.0 g). There was a significant positive correlation (ρ = 0.778, *P* < 0.001) between the stool volume assessed by the participants and the actual measured weight of the faeces (Fig. 3a). Comparison of the researcher’s observed stool volume and the actual measured weight revealed a better correlation (ρ = 0.940, *P* < 0.001) than that of the participants. A regression equation (y = 19.3x + 9.2) was calculated from the stool volume evaluated by each participant (x) and the actual measured weight (y). The estimated faecal weight per stool model unit calculated using the regression equation was 47.8 g.

#### Stool form

The participants’ responses on stool form based on the faecal assessment tool were distributed between Types 2 and 7, with Type 4 being the most common response (42.1%). The normal stool forms, Type 3 (23.7%), Type 4 (42.1%), and Type 5 (13.2%), accounted for 79% of the responses (Table 1). There were no responses for Type 1 and only one participant reported Type 7 (2.6%). The faecal moisture content percentage was 76.9% ± 7.1% (range, 64.4%–90.2%). A significant positive correlation was observed between the participant-observed stool form responses and the faecal moisture content percentage (ρ = 0.717, *P* < 0.001) (Fig. 3b). The correlation (ρ = 0.782, *P* < 0.001) was stronger between the researcher’s observed stool form responses and the faecal moisture content percentage.

The average faecal hardness evaluated using the Creep Meter was 1.68 ± 1.7 (N) (range, 0.07–6.2). The average faecal adhesiveness was 2,696 ± 2,481 J/m^3^ (range, 87.6–10,185). A significant negative correlation (ρ = −0.843, *P* < 0.001) was observed between the participants’ observed stool forms and the physical measurement of the faecal hardness (Fig. 3c). A significant negative correlation (ρ = −0.761, *P* < 0.001) was also observed between the participant’s responses and the adhesiveness of the faeces. Compared to that of the participants, the correlation was stronger between the researcher’s responses and the stool hardness (ρ = −0.902, *P* < 0.001) and adhesiveness (ρ = −0.816, *P* < 0.001).

#### Stool colour

Observational judgment using the faecal assessment tool was compared to objective colour evaluation using a colourimeter. The participants’ observational colour judgment responses were as follows: colour 3, 28.9%; colour 4, 55.3%; and colour 5, 15.8%. None of the participants reported colours 1, 2, or 6 (Table 1).

**Figure 4:**
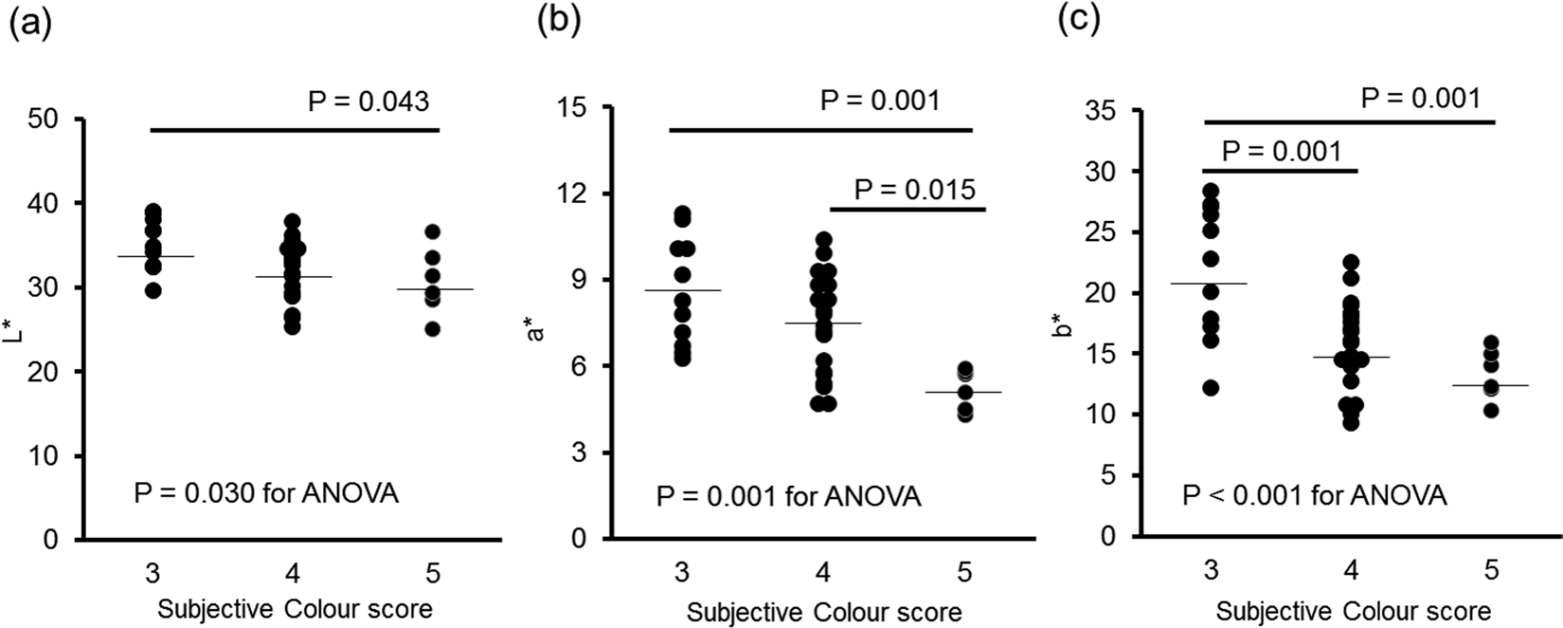
Relationship between stool colour by observational evaluation and L*a*b* values determined using a colourimeter. The black dots indicate individual values, while the bars indicate the average values (*n* = 38). The data were analysed by one-way ANOVA and the differences among the mean values were analysed using Tukey’s post hoc test. Colour values: (**a**) L*; lightness (**b**) a*; green-red (**c**) b*; blue-yellow.

The mean values of the three indices used to objectively evaluate faecal colour were: L*-value 32.8 ± 3.6 (range, 25.1–39.0), a*-value 7.4 ± 2.0 (range, 4.3–11.3), and b*-value 17.1 ± 5.1 (range, 9.0–28.4). There were significant differences in L*a*b* values among the colour responses using the faecal assessment tool. A significant difference in L* value indicating lightness was observed between colour 3 (yellowish-brown) and colour 5 (greenish-dark brown) (35.1 ± 3.1 vs. 30.7 ± 4.0, respectively, *P* = 0.043, Fig. 4a). Significant differences in the a* and b* values indicating redness and yellowness were observed between colour 3 and colour 5 (8.6 ± 1.8 vs. 5.2 ± 0.7, *P* = 0.001; 21.9 ± 5.5 vs. 13.3 ± 2.1, *P* = 0.001, respectively, Fig. 4b,c). Furthermore, the researcher’s evaluations were similar to those of the participants, and also differed significantly compared to the objective index.

#### Stool odour

The objectively evaluated Odour Index yielded a mean value of 391 ± 172 (range, 146–821). There was no significant correlation between the observational odour evaluation (four levels) and the Odour Index (ρ = −0.048, *P* = 0.777). However, there was a significant positive correlation between the researcher’s observational odour evaluation and the Odour Index (ρ = 0.694, *P* < 0.001).

### Examination of the degree of coincidence between observers

Significant correlations were observed between the participants’ and the researcher’s evaluations with regard to the stool volume (ρ = 0.793, *P* < 0.001), form (ρ = 0.874, *P* < 0.001), and colour (ρ = 0.726, *P* < 0.001), as shown in Table 2. There was no significant correlation for stool odour (ρ = 0.221, *P* = 0.182). Moreover, moderate degrees of coincidence, evaluated using the kappa coefficient, were observed for stool volume (weighted κ = 0.708, *P* < 0.001) and colour (weighted κ = 0.708, *P* < 0.001). Furthermore, there was a high degree of coincidence for stool form (weighted κ = 0.887, *P* < 0.001), but a low degree of coincidence for stool odour (weighted κ = 0.243, *P* = 0.031).

**Table 2:** Degree of coincidence between observational evaluation of each participant (*n* = 38) and a researcher using the faecal assessment tool.

	Spearman’s rho	*P*-value	Weighted κ	*P*-value
Stool volume	0.793	<0.001	0.708	<0.001
Stool form	0.874	<0.001	0.887	<0.001
Stool colour	0.726	<0.001	0.708	<0.001
Stool odour	0.221	0.182	0.243	0.031

Interpretation of Kappa (κ) values given by Landis and Koch (1977). Kappa Agreements were: <0.20, slight; 0.21–0.40, fair; 0.41–0.60, moderate; 0.61–0.80, substantial; and ≥0.81, almost perfect.

## Discussion

To evaluate the validity of a faecal assessment tool to assess faecal conditions, the observational assessments of stool volume, form, colour, and odour were compared to objective and physical characteristics of the actual faeces in healthy adults. The results can be summarized as follows. (1) The number of stool model units and the faecal weight measured using the weighing scales showed a strong and significant correlation. (2) A moderate significant positive correlation was observed between the observational stool form using the seven-level BSFS and the moisture content. Moderate-to-high significant negative correlations were observed for the observational stool form and faecal hardness and adhesiveness. (3) There were significant differences in the L*a*b* colour values measured using the colourimeter among the observational colour responses in the faecal assessment tool. (4) There was no significant correlation between the observational odour evaluation and the odour index. (5) The correlations between the observational assessments and objective results by the researcher were stronger than those made by each participant. (6) The degrees of coincidence of each participant’s observational evaluations and the researcher’s observational evaluations showed moderate degrees of coincidence for stool output and colour, a high degree of coincidence for stool form, and a low degree of coincidence for stool odour. These results suggest that it is possible to effectively estimate volume, form, and colour, but not odour, of stool using the faecal assessment tool.

The BSFS is one of the most widely used indices in both clinical and research settings^28^ for assessing faecal condition based on faecal from and apparent texture. However, there are no data on the validity or reliability of faecal evaluation considering not only stool form but also volume, colour, and odour, simultaneously. Here, we developed an easy to use tool that allows immediate visual comparison and observation of the actual faeces. The tool is the size of a postcard and consists of an actual-size cylindrical model for faecal volume, a modified form model based on the BSFS, and a colour scale for evaluating the stool colour, as seen in Fig. 1. Moreover, a 10-cm length scale at the edge of the card allows easy measurement of the actual faeces. This study demonstrated the validity of this multifaceted faecal assessment tool that combines several faecal condition evaluations.

Faecal weight has been reported to be associated with the level of dietary fibre intake^14,42^ and the risk of developing colon cancer^14^, which has been reported to be lower in cases with daily faecal volume >150 g^14^. Therefore, it is important to observe day-to-day stool volume. A life-size representation of a cylindrical model was used to assess stool volume, and the participants responded according to the number of faecal model units. As there was a strong positive correlation between each participant’s observed stool volume and the faecal weight, the observed stool volume reflects the actual measurement of the faecal weight. The estimated faecal weight per unit calculated using the regression equation of the actual faecal weight (y) and the observed stool volume (x) was 47.8 g. This result is useful for the conversion of stool model units into numerical values to determine faeces weight. However, the stool weight estimated using this conversion equation may not precisely convert various stool forms with different densities, such as solid and loose/liquid. Therefore, further research is needed to allow estimation of absolute values.

The moisture content of faeces gradually increased as the stool form according to the BSFS became more watery. Blake *et al*. reported a significant correlation between stool form classified by specialists using the BSFS and faecal moisture content (ρ = 0.701, *P* < 0.001), which was more strongly correlated than that of volunteers (ρ = 0.491, *P* < 0.001)^37^. The present study yielded similar results, except that the faecal evaluation by each participant in this study showed a stronger correlation (ρ = 0.717, *P* < 0.001). Moreover, there were significant negative correlations between the observational evaluation of the stool form using the BSFS and the stool hardness or adhesiveness measured using the Creep Meter. There was also a high degree of coincidence in the observational stool form evaluation between each participant and the researcher. These findings suggested that the observational evaluation of stool form using the BSFS reflects the physical properties of faeces, including moisture content, hardness, and adhesiveness, and that it is possible for individuals inexperienced with the BSFS to also observationally evaluate their stool form.

Stool colour is affected by daily diet and by the secretion of digestive fluids, such as bile, and has also been reported to be associated with the occurrence of bile duct disease and colon cancer^34,43^. There were significant differences in the L*a*b* colour space evaluation results using the colourimeter among the observational colour responses, suggesting a discriminative ability to detect differences in stool colour using the faecal assessment tool. In this study, the colours selected by the participants ranged within 3–5 on the faecal assessment tool, and the colours at both extremes were not selected. Colours 1, 2, and 6 from the faecal assessment tool may not have been applicable to any of the faecal samples likely due to the inclusion of healthy participants in the study. In addition, there were no samples with L*a*b* colour values that were outliers in the objective colour evaluation. To evaluate the validity of this faecal assessment tool, it is necessary to examine the tool in a larger population. Moreover, a more detailed classification including further subdivision of colours 3–5 may be needed for healthy individuals. Conversely, a moderate degree of coincidence was observed between each participant’s observational evaluation and the researcher’s observational evaluation using the colour of the faecal assessment tool, suggesting that this tool can be used by individuals lacking specialized knowledge in the field. Therefore, objective colour evaluation using the L*a*b* colour space was suggested as a reliable method to quantify faecal colour. With further study, this method could become a simple and effective means to assess the effects of faecal colour management.

The observation of stool odour has been used to diagnose gastrointestinal diseases and to evaluate bowel conditions^23,44^. In the present study, there were no significant correlations between the observational odour evaluation by each participant using the faecal assessment tool and the odour intensity index calculated using the odour measuring device. The observational odour evaluation produced different results from volume, form, and colour assessments because there was no reference control for odour. Moreover, the evaluation of odour was based on the participant’s empirical and relative senses, and the odour measured by the device measured the overall odour intensity of the faeces without specifying the odour that made people feel uncomfortable. Therefore, the observation of stool odour should be removed from the present tool to refine its overall accuracy. The card tool should be used to evaluate volume, shape, and colour of feces in a manner comparable to visual references.

To date, many studies on the observational evaluation of faeces have been performed in both patients and healthy subjects, but the present study population included only healthy Japanese people. External validation will be necessary in subjects of other ethnicities, languages, regions, age groups, socioeconomic and health status, etc. Moreover, the observations using the faecal assessment tool were performed on a white tray in the present study. As most users will commonly observe their faeces in a toilet bowl, it is possible that the accuracy of evaluation under real-world conditions will be lower than in this study. The strength of this study was that we were able to distinguish faecal conditions even in a small group of healthy individuals in whom faecal conditions were not diverse. Furthermore, faecal assessment tools used in previous studies have only focused on one aspect of faecal condition^22,29,37^. Our tool allowed multifaceted observations of three different aspects of faecal condition, i.e., volume, form, and colour.

Previous studies revealed a negative correlation between faecal weight and the risk of developing colon cancer^3,14^. Faecal form assessed by BSFS has also been reported to be associated with a number of gastrointestinal diseases^4,16–20^. Stool colour is also associated with biliary atresia in infants^21^, and upper and lower gastrointestinal bleeding^22^. In addition, several studies indicated that faecal volume and form were associated with dietary fibre intake^27^ and gut microbiome enterotype^13^. Therefore, it is possible that the multifaceted assessment of stool can be used to classify patients according to disease subtype or degree of progression, and it will provide useful information for screening or diagnosis. Moreover, it can contribute to more accurate assessment of daily dietary intake or nutrient status, because self-reported dietary intake has been reported to be underestimated in many cases. While many previous studies have demonstrated the usefulness of understanding faecal condition, these studies were limited by their evaluation of only one aspect of faecal condition or by small sample size. Thus, multifaceted evaluation of faecal condition, especially in large-scale studies, will be required to achieve more accurate screening of various diseases and to gain a better understanding of the status of an individual’s diet and nutrition.

We have designed a card tool that enables the self-assessment of stool volume, form, colour, and odour, and compared the observations using this tool with objective evaluations. Satisfactory correlations were observed between the observational and objective evaluations of volume, form, and colour of faeces obtained from healthy adults, suggesting that the illustrative tool addressing volume, form, and colour of faeces can be used to comprehensively assess faecal condition. Further research is necessary for the faecal assessment tool to be useful in screening for diseases or to improve understanding of human health and nutritional conditions.

## References

[1] Leser TD, Molbak L (2009). Better living through microbial action: the benefits of the mammalian gastrointestinal microbiota on the host. Environ. Microbiol..

[2] Woodmansey EJ (2007). Intestinal bacteria and ageing. J. Appl. Microbiol..

[3] Cummings JH, Bingham SA, Heaton KW, Eastwood MA (1992). Fecal weight, colon cancer risk, and dietary intake of nonstarch polysaccharides (dietary fiber). Gastroenterology.

[4] Palsson OS, Baggish JS, Turner MJ, Whitehead WE (2012). IBS patients show frequent fluctuations between loose/watery and hard/lumpy stools: implications for treatment. Am. J. Gastroenterol..

[5] Van den Abbeele P, Van de Wiele T, Verstraete W, Possemiers S (2011). The host selects mucosal and luminal associations of coevolved gut microorganisms: a novel concept. FEMS Microbiol. Rev..

[6] Cani PD, Delzenne NM (2011). The gut microbiome as therapeutic target. Pharmacol. Ther..

[7] Baothman OA, Zamzami MA, Taher I, Abubaker J, Abu-Farha M (2016). The role of Gut Microbiota in the development of obesity and Diabetes. Lipids Health Dis..

[8] Gagniere J (2016). Gut microbiota imbalance and colorectal cancer. World J. Gastroenterol..

[9] Brunkwall L, Orho-Melander M (2017). The gut microbiome as a target for prevention and treatment of hyperglycaemia in type 2 diabetes: from current human evidence to future possibilities. Diabetologia.

[10] David LA (2014). Diet rapidly and reproducibly alters the human gut microbiome. Nature.

[11] Wu GD (2011). Linking long-term dietary patterns with gut microbial enterotypes. Science.

[12] Tremaroli V, Backhed F (2012). Functional interactions between the gut microbiota and host metabolism. Nature.

[13] Vandeputte D (2016). Stool consistency is strongly associated with gut microbiota richness and composition, enterotypes and bacterial growth rates. Gut.

[14] Birkett AM, Jones GP, de Silva AM, Young GP, Muir JG (1997). Dietary intake and faecal excretion of carbohydrate by Australians: importance of achieving stool weights greater than 150 g to improve faecal markers relevant to colon cancer risk. Eur. J. Clin. Nutr..

[15] Mearin F (2016). Bowel Disorders. Gastroenterology.

[16] Probert CS, Emmett PM, Cripps HA, Heaton KW (1994). Evidence for the ambiguity of the term constipation: the role of irritable bowel syndrome. Gut.

[17] Heaton KW, Ghosh S, Braddon FE (1991). How bad are the symptoms and bowel dysfunction of patients with the irritable bowel syndrome? A prospective, controlled study with emphasis on stool form. Gut.

[18] Thurmon KL, Breyer BN, Erickson BA (2013). Association of bowel habits with lower urinary tract symptoms in men: findings from the 2005-2006 and 2007-2008 National Health and Nutrition Examination Survey. J. Urol..

[19] Malhotra A (2016). Use of Bristol Stool Form Scale to predict the adequacy of bowel preparation - a prospective study. Colorectal Dis..

[20] Choung RS, Locke GR, Zinsmeister AR, Schleck CD, Talley NJ (2007). Epidemiology of slow and fast colonic transit using a scale of stool form in a community. Aliment. Pharmacol. Ther..

[21] Chen SM (2006). Screening for biliary atresia by infant stool color card in Taiwan. Pediatrics.

[22] Zuckerman GR, Trellis DR, Sherman TM, Clouse RE (1995). An objective measure of stool color for differentiating upper from lower gastrointestinal bleeding. Dig. Dis. Sci..

[23] Garner CE (2007). Volatile organic compounds from feces and their potential for diagnosis of gastrointestinal disease. FASEB J..

[24] Ahmed I, Greenwood R, Costello Bde L, Ratcliffe NM, Probert CS (2013). An investigation of fecal volatile organic metabolites in irritable bowel syndrome. PLoS One.

[25] Sonoda H (2011). Colorectal cancer screening with odour material by canine scent detection. Gut.

[26] de Boer NK (2014). The scent of colorectal cancer: detection by volatile organic compound analysis. Clin. Gastroenterol. Hepatol..

[27] Burkitt DP, Walker A, Painter NS (1972). Effect of dietary fibre on stools and transit-times, and its role in the causation of disease. The Lancet.

[28] Lewis SJ, Heaton KW (1997). Stool form scale as a useful guide to intestinal transit time. Scand. J. Gastroenterol..

[29] Whelan K, Judd PA, Taylor MA (2004). Assessment of fecal output in patients receiving enteral tube feeding: validation of a novel chart. Eur. J. Clin. Nutr..

[30] Whelan K, Judd PA, Preedy VR, Taylor MA (2007). Enteral feeding: the effect on faecal output, the faecal microflora and SCFA concentrations. Proc. Nutr. Soc..

[31] Lien TH (2011). Effects of the infant stool color card screening program on 5-year outcome of biliary atresia in Taiwan. Hepatology.

[32] Schreiber RA (2014). Home-based screening for biliary atresia using infant stool colour cards: a large-scale prospective cohort study and cost-effectiveness analysis. J. Med. Screen..

[33] Gu YH (2015). Stool color card screening for early detection of biliary atresia and long-term native liver survival: a 19-year cohort study in Japan. J. Pediatr..

[34] Shen Z, Zheng S, Dong R, Chen G (2016). Saturation of stool color in HSV color model is a promising objective parameter for screening biliary atresia. J. Pediatr. Surg..

[35] Heaton K (1992). Defecation frequency and timing, and stool form in the general population: a prospective study. Gut.

[36] Aichbichler BW (1998). A comparison of stool characteristics from normal and constipated people. Dig. Dis. Sci..

[37] Blake MR, Raker JM, Whelan K (2016). Validity and reliability of the Bristol Stool Form Scale in healthy adults and patients with diarrhoea-predominant irritable bowel syndrome. Aliment. Pharmacol. Ther..

[38] Katsuki A, Fukui KH (1998). 2 selective gas sensor based on SnO 2. Sensors and Actuators B: Chemical.

[39] Faul F, Erdfelder E, Lang AG, Buchner A (2007). G*Power 3: a flexible statistical power analysis program for the social, behavioral, and biomedical sciences. Behav. Res. Methods.

[40] O’Donnell L, Virjee J, Heaton KW (1990). Detection of pseudodiarrhoea by simple clinical assessment of intestinal transit rate. BMJ: British Medical Journal.

[41] Landis JR, Koch GG (1977). The measurement of observer agreement for categorical data. Biometrics.

[42] de Vries J, Birkett A, Hulshof T, Verbeke K, Gibes K (2016). Effects of Cereal, Fruit and Vegetable Fibers on Human Fecal Weight and Transit Time: A Comprehensive Review of Intervention Trials. Nutrients.

[43] Chen K (2003). Nested case-control study on the risk factors of colorectal cancer. World J. Gastroenterol..

[44] Poulton J, Tarlow MJ (1987). Diagnosis of rotavirus gastroenteritis by smell. Arch. Dis. Child..

